# Multi-Indicator Approach for Characterising Urban Green Space Provision at City and City-District Level in Germany

**DOI:** 10.3390/ijerph16132300

**Published:** 2019-06-28

**Authors:** Karsten Grunewald, Benjamin Richter, Martin Behnisch

**Affiliations:** 1 Leibniz Institute of Ecological Urban and Regional Development, Weberplatz 1, 01217 Dresden, Germany; 2 State Capital of Dresden, Environmental Office, P.O. Box 120020, D-01001 Dresden, Germany

**Keywords:** ecosystem service, green characteristic, green space access, grey characteristic, hemeroby, settlement density, soil sealing

## Abstract

This paper addresses the question of how a sustainable urban development can be supported through simple measured quantities in the context of the specific provision of green space and open space. The specific provision of green space is analysed based on a combination of six indicators that describe, on the one hand, the access of inhabitants to green spaces and on the other hand, the settlement character as well as the strong anthropogenic imprint on the urban landscape. The indicators were calculated and combined in a 9-cell matrix for classifying the areas studied. The implementation was carried out at two scales for all German cities with at least 50,000 inhabitants as well as exemplarily for city districts of eight big cities. The calculated indicator values for representing green characteristics decrease with increasing number of inhabitants, whereas the opposite relationship was obtained for the indicators of the grey characteristics. We show how the approach provides an in-depth morphological assessment of German cities ranking their scores from low to the high presence of grey/green characteristics.

## 1. Introduction

In the past decades, the land use development in many parts of the world has mostly been characterised by a steady increase in settlement and transportation areas and is contrary to the principles of sustainability [1,2,3,4,5,6]. The causes are quite complex and land consumption can often only be explained by a bundle of influencing factors [7,8].

There is a rising demand for residential space per capita, an increase in numbers of inhabitants due to migration, as well as the designation of industrial and commercial areas in the open space, sometimes despite existing urban fallow and converted areas, not only in Germany [9,10,11]. The progressive urbanisation at the expense of the open space, negative changes in urban climate, and the adaptation to a changing climate require the protection and development of both inner-city and peripheral green spaces as balancing spaces to ensure ecosystem services that are essential for the health and safety of its inhabitants [12,13,14,15]. At the national political level in Germany, important measures and strategies for promoting sustainable urban development in the context of green spaces include:

– promotion of building development in the inner area of settlements (§ 35 BauGB (Federal Building Code))
– urban development funding programme ‘Zukunft Stadtgrün’ (‘Future Urban Green’), protection and development of functional urban ecosystems and their performance capacity [11,16]
– reduction of the land consumption for settlement and transportation uses [17,18]

The promotion of building development in the inner area of the cities can lead to a local decrease in green spaces and also to a reduced interconnection of green spaces [11,16]. As a consequence, conflicts of interest with respect to the German sustainability strategy (reduction of land consumption, [17]) and the biodiversity strategy (enhanced greening in cities, [19]) increase with densification.

A sustainable urban development strives for an optimal relationship between grey and green characteristics in order to ensure the respective functionalities. In particular, buildings and streets that provide public service functions (e.g., living, working, learning) are part of the grey areas. Spaces characterised by vegetation (e.g., green spaces, urban forests) and bodies of water form the green characteristics, which makes a contribution to biodiversity and provides multiple ecosystem services such as air filtering, recreation, flood retention (e.g., [12,14,15,20,21,22,23]). At the international level also a considerable number of studies investigating well-being and health benefits exist. For an overview see Wüstemann and Zhang [24]. Urban green space positively influences life satisfaction in general [25,26].

Besides restorative effects in mental health and increasing physical activities, the mechanisms through which green space benefits health also include improving air quality, including through removing ozone [27] and storing carbon dioxide [28,29], buffering anthropogenic noise [30], decreasing the urban heat island effect, improving the immune system through microbial input from the environment to drive immunoregulation [31], and fostering social cohesion [32].

The provision of a city with green space is also significant for its image as an attractive place worth living in. Thus, the provision of green space is a location factor when cities compete for tourists and for attracting businesses that want to offer their employees a residential environment worth living in. However, in a society with an increasing concentration of population in big cities, the demand for a comprehensive provision of inhabitants with green space often conflicts with the necessary creation of new residential space, e.g., in form of supplemental developments between buildings or on vacant spaces. The concept of a compact city [33,34,35] requires an integrative view to approach ecologically and environmentally sustainable development which reflects trade-offs that occur mainly between densification and the quantitative and qualitative supply of green spaces within urban developed areas. Such an integrative view is important specifically in city ecosystem services, and thus urban green spaces as important suppliers of ecosystem services, need to be provided where they are in demand [20,36,37].

Urban spaces are explicitly included in the framework of the EU strategy on ‘Green Infrastructure’ [38]. The German federal strategy ‘Green Infrastructure’ deals with urban green spaces programmatically but ‘does not represent them concretely in space and cartographically for reasons of scale’ [39]. Deriving goals for the development of concrete urban green infrastructure in the context of construction projects in existing settlements requires statements backed by measurable quantities. The White Paper Urban Green [13] names quantitative and qualitative measures for the green and primary unsealed open space development that are to be developed: accessibility of green space, provision of green space, green quality and green space factor. They are meant to serve for formulating standards of ‘adequate provision of open space’ of the inhabitants with urban green and primary unsealed open spaces [40,41]. However, the value of the characteristic quantities can vary widely depending on the database used (source and selection of land use types, census) and the thresholds set (e.g., distances, area requirements).

A report on green space to support healthy living in urban areas was provided by the World Health Organization [42]. It presents a suggested indicator of accessibility of green space with examples of its application in three European cities and a detailed methodological tool kit for Geographical Information System (GIS) analysis of land use and population data. Dosch and Neubauer [40] developed characteristic values for urban greenery as examples for the city of Vienna, which have been incorporated into the international indicator debate. A key defining feature of green space measures used in health research and policy is whether they consider the availability, accessibility or usage of green spaces [42].

In this context, this paper addresses the question of how a sustainable urban development can be supported through measured quantities in the context of green space and open space planning. The specific provision of green space is analysed based on a combination of six indicators that describe, on the one hand, the access of inhabitants to green spaces and, on the other hand, the settlement character as well as the strong anthropogenic imprint on the urban landscape.

We use a classification approach for each indicator based on points according to the rank of a city. The results are presented and discussed at two spatial scales (entire city and city districts) using the example of big German cities. The indicators are intended to be updatable (suitable for monitoring), but due to data availability and rising model complexity, changes over time are not treated in depth in the paper.

Taking into consideration potential trade-offs between making the framework complex and keeping it simple as well as providing meaningful insights from a scientific and practical point of view is challenging. In this respect, the combined indicator approach for characterising cities/city districts with 3 sub-indicators for ‘green’ and 3 for ‘grey’ characteristics that are discussed in the paper differs from even more complex approaches such as the ‘Green Cities Index’. The Green City Index methodology was developed by the Economist Intelligence Unit (EIU) in cooperation with Siemens and measures cities regarding to their environmental performance on approximately 30 indicators across eight to nine categories depending on the region [43]. Our multi-indicator approach is similar to the multiple scenario modeling approach for the region of Munich, Germany, by Xu et al. [44]. They developed multiple scenarios with respect to three dimensions: housing demand (high, medium or low), urban spatial structure (monocentric or polycentric) and urban growth form (sprawl, compact sprawl or compact).

## 2. Measurement Approach

In practice, mostly indicators such as ‘accessibility of urban green space’ (mainly in the residential environment reachable by foot) and ‘green space per inhabitant’ are used as quantitative measures (indicators) for characterising the provision of the population with public green spaces in the urban space [40,45,46,47,48,49]. For assessing the cultural ecosystem service ‘recreation in the city’ Grunewald et al. [50] developed the indicator ‘accessibility of green space’. In this context, the presence of green areas for all German cities with more than 50,000 inhabitants was mapped and combined with residential areas to assess, if the ecosystem service supply matches the demand for after work recreation. This indicator was intensively discussed with experts and practitioners and is politically accepted and proposed for the German Biodiversity Strategy [50].

Complementarily, quantities are established that tend to represent the grey characteristics, such as ‘settlement density’ or ‘soil sealing’ [51]. The strong anthropogenic imprint on ecosystems can also be assessed in the urban space through the ‘degree of human influence’, measured as a sum indicator through the hemeroby approach [52,53]. This reflects important aspects of the human imprints on ecosystems [54,55]. Grey characteristics serve to satisfy central human basic needs or basic functions of existence as living, working, caring, education, recreation, traffic participation, and socio-cultural activities. In order to ensure good living conditions and human well-being, a balance must be achieved between providing basic human needs through grey characteristics and environmental functions or ecosystem services through green characteristics [54].

In the following, a combination of six measures is proposed in order to characterise all German cities with at least 50,000 inhabitants and, exemplarily, city districts of eight big cities in their specific provision of green space and open space. Relevant indicators were selected that are available at the federal level or are easy to acquire and updatable (Section 2.1; Table 1). The indicators are calculated individually and subsequently combined in the framework of a classification.

### 2.1. Short Description of the Indicators and Data Used

The indicator *(i1) ‘accessibility of urban green space’* captures the population that can reach both green spaces ≥ 1 ha within walking distance (accessibility of nearby green spaces) and green spaces ≥ 10 ha at medium distance (accessibility of larger green spaces at a medium distance) and places it in relation to the total population. For more methodological explanations, e.g., how the data about recreational areas were selected, see Grunewald et al. [50]. In general, the approach is comparable to the recommendations of the WHO [48] for conceptualisation and measurement of indicators of urban green space availability, accessibility and usage as well as assessment of their health relevance.

The provision of green space per inhabitant was implemented in two forms. On the one hand, *(i2) ‘green space provision—settlement’* captures all green spaces with recreational function (without consideration of municipal borders) in the vicinity (300 m) of predominantly inhabited and contiguously built-up areas and relates them to the number of inhabitants of the respective municipality. On the other hand, *(i3) ‘green space provision—total’* determines all green spaces with recreational function within a municipality and relates them to the total number of inhabitants [50].

Generally, in our approach higher indicator values i1, i2, and i3 indicate that the provision with green space is better ensured in the area under consideration.

For describing grey areas (or the provision of open space), our measurement approach draws on the indicator *(i4) ‘soil sealing—settlement’*, which describes the proportion of the area in which the soil is covered or sealed by partly permeable (e.g., grass pavers) or impermeable materials (e.g., concrete, asphalt). The indicator *(i5) ‘settlement density’* measures the number of inhabitants in relation to the inhabited settlement area (Table 1). This input quantity used in our measurement approach slightly differs from the indicator defined in the German Sustainability Strategy [18], as the number of inhabitants is referred only to the inhabited settlement area and not to the total settlement area. The indicator *(i6) ‘hemeroby’* measures the distance between the current vegetation and an assumed final state of self-regulated vegetation in the complete absence of human intervention [52,53].

For the indicators i4, i5 and i6, we assume in our measurement approach that the area analysed is more strongly characterised by grey areas the more sealed it is, the denser the settlement area is and the less it is close to nature [44,55].

ATKIS Basis-DLM (Digital Landscape Model) is the central data base for the national ecosystem services assessment in Germany [56]. It contains nationwide information on land use with a high thematic and spatial resolution and quality [57,58]. For the land use types, the lower limit of data acquisition is 1 ha, but some of them are acquired completely (e.g., sports and leisure facilities, game parks). The update is carried out cyclically for all areas after at most 3–5 years, using aerial photography and a multitude of thematic details.

The land use data of the European Urban Atlas (EUA) represent an alternative to the Basic-DLM (www.eea.europa.eu/data-and-maps/data/urban-atlas). They are particularly suitable for European comparisons (e.g., [47,48]). The EUA includes categories relevant to the indicators to be calculated (‘Forest’, ‘Water’), but also some highly aggregated categories such as ‘Green urban areas’ or ‘Agricultural’. A number of big cities in Germany such as Münster, Mannheim, and Chemnitz (data set 2006) as well as Dresden and Magdeburg (data set 2012) are not represented in the EUA. For these reasons, but also because the Basic-DLM has a higher thematic and temporal resolution, the ATKIS Basic-DLM is preferred for the development of indicators in Germany [50,56].

Socio-economic data, such as inhabitant data, are another important information base, which represents the ‘demand’ of ecosystem services in general. For instance, inhabitants are taken into account as potential consumers of the ecosystem service ‘recreation in the city’ [49,50], drawing on census data or the municipal directory information system. The German census data are updated only every 10 years, which limits the monitoring.

Grid data on hemeroby and soil sealing from 2012 published in the IOER Monitor (www.ioer-monitor.de/home/?L=1) are additionally taken into account in the analysis. In addition, geometries are required as reference points for the calculation of indicators in the geographic information system (GIS). Data used in our analysis are listed in the following:

– *ATKIS Basis DLM: data set 2015*, source: Federal Agency for Cartography and Geodesy (Bundesamt für Kartographie und Geodäsie—BKG)
– *Hemeroby 2012*: indicator Hemeroby 2012 (grid 100 m), source: IOER Monitor, own calculations
– *Imperviousness 2012*: Indicator Imperviousness 2012 (grid 100 m), source: IOER Monitor, own calculations based on Imperviousness Data 2012 of the European Environment Agency
– *Administrative city boundaries* (municipal boundaries): administrative boundary geometry VG25, source: BKG
– *Administrative city district boundaries* (one level below municipal boundaries): OSM Boundaries Map 4.1, source: OpenStreetMap (https://osm.wno-edv-service.de/boundaries/)
– *Polygon features of basic raster geometries* (INSPIRE grid 100 m), source: IOER, own calculations
– *Population raster of the 2011 census, size of raster cells 100 m*, source: Federal Statistical Office (Statistisches Bundesamt, DESTATIS)
– *The municipal directory information system* (Gemeindeverzeichnis-Informationssystem), source: Federal Statistical Office (Statistisches Bundesamt, DESTATIS).

### 2.2. Ranking and Classification via 9-Cell Matrix

**Figure 1:**
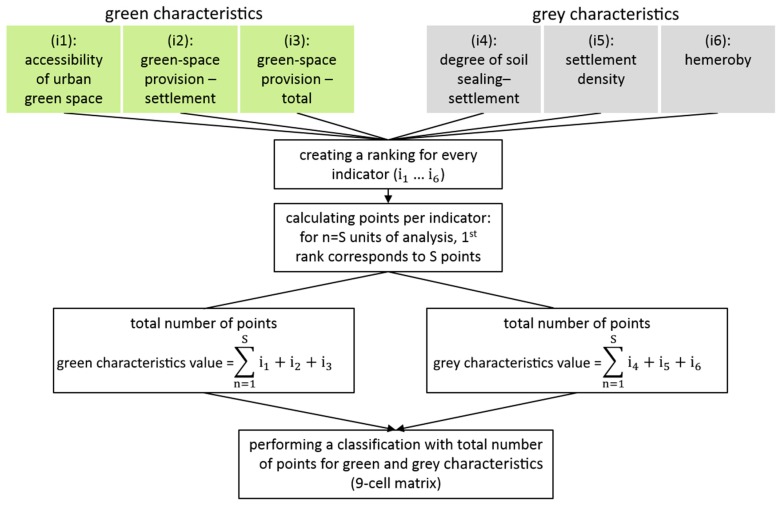
Scheme of the rank-based classification for characterising green space provision of cities using a combined indicator (modified according to Richter et al. [59]).

**Figure 2:**
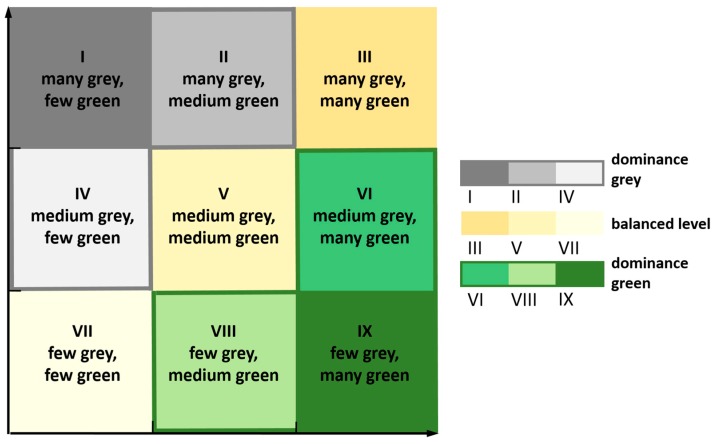
The ‘9-cell matrix’ as the basis for the classification.

In the present contribution, a classification based on points awarded according to rank is used for characterising the areas studied with respect to the provision of the inhabitants with green space. In the approach, the indicators (i1)—(i6) described above are first calculated, and subsequently the areas studied are sorted in descending order according to the indicator values determined (Figure 1). In the next step, a number of points is derived using the reverse order. The number of points awarded for the first rank coincides with the total number of cities studied. All units of analysis receive a number of points for each indicator. The awarded points are summed separately for grey and green areas in cities. Subsequently, the summed values are entered into a 9-cell matrix with two coordinate axes (Figure 2) for a portfolio analysis. Depending on the cell assignment, the corresponding unit of analysis exhibits a dominance of grey or of green areas or a balanced level.

## 3. Results

### 3.1. Results of the Individual Indicators i1–i6

The calculation of the indicator values, carried out for all German cities from 50,000 inhabitants upwards with the same data basis and calculation steps, allows for a comparative characteristic at the city level (statistics in Table 2, reference year 2015). In addition to the overall perspective on all cities (*n* = 187), 3 classes of city size and their measurements are identified: cities > 500,000 inhabitants (‘big city’, *n* = 13), cities > 100,000–500,000 inhabitants (‘city’, *n* = 64), cities ≥ 50,000–100,000 inhabitants (‘town’, *n* = 110).

The mean accessibility of urban green space (i1) amounts to approx. 80% of the German city population. The cities of Bayreuth, Dinslaken, Aschaffenburg, Dorsten or Siegen are representatives (median = 80.4). Cities with a particularly good accessibility of green space include some towns and medium-sized cities such as Wetzlar (99.1), Gießen (98.5), Schwerin (97.7), Ulm (96.0) or Bonn (95.4). 50% of German cities exhibit a value between 71.05 (Q_1_) and 87.55 (Q_3_). Norderstedt (min = 49.5), Erfurt (52.9) or Hanau (53.3) have particularly low values.

The cities of Viersen or Aschaffenburg (median = 128.7) are characteristic for the provision of green space in the settlement area (i2). Gummersbach (max = 902.4) is an extreme positive example. It is followed by Stolberg (447.7), Passau (410.5) and Siegen (405.4). Cities with more than a million inhabitants like Munich (min = 36.1) or Berlin (41.9) but also Frankfurt am Main (36.5) exhibit rather low values for this indicator.

With 46.1 m^2^ per inh., the city of Munich also represents the minimum with respect to the total provision of urban green space (i3), followed by Herne (74.0) and Ludwigshafen (83.6). The cities of Bottrop (median = 346.8), Aachen (353.4) or Heidelberg (345.5) represent typical examples of green-space provision. The less densely built-up cities of Arnsberg (max = 1942.2), Brandenburg an der Havel (1926.5) or Baden-Baden (1924.4) are extreme positive examples.

Cities with a particularly good supply with respect to the indicators i1 to i3 include Dessau-Roßlau, Wetzlar, Wesel, Goslar, Passau, Schwerin, Kaiserslautern, Brandenburg an der Havel or Baden-Baden. By contrast, big cities such as Frankfurt am Main, Leipzig, Mannheim, Krefeld, Berlin or Hanover exhibit a lower supply. Concerning green-space provision (i2, i3), one predominantly finds that the smaller the cities, the higher the green-space provision. On the other hand, the mean accessibility of urban green space (i1) is relatively similar for all classes of city size.

The value range of the proportion of sealed area relative to the inhabited settlement area (i4) lies between 23.4% and 66.9% in German cities. In the mean, nearly 50% of inhabited settlement areas are sealed. Typical examples include the cities of Reutlingen (median = 48.4), Konstanz (48.3), Bonn (48.3), Mülheim an der Ruhr (48.5) or Wolfsburg (48.8). In Germany, Rüsselsheim (max = 66.9) has the highest proportion of sealed area in the settled areas, and Gummersbach (min = 23.4) the lowest.

The mean settlement density (i5) is 148.6 inhabitants per hectare of predominantly inhabited settlement area. Cities in the Rhine-Main metropolitan region exhibit the highest values of settlement density: e.g., Frankfurt am Main (max = 317), Mannheim (291), Offenbach (290), Ludwigshafen (277). Minden (min = 73), Gummersbach (73), Detmold (74) or Celle (76) are examples of less densely built-up cities. The typical settlement density amounts to 138 inh./ha (median) and is represented by the cities of Leverkusen, Düren, Gladbeck or Landshut.

The values of hemeroby (i6) in German cities with ≥50,000 inhabitants vary between 3.5 (moderately affected by culture, for example, Baden-Baden) and 5.5 (very strongly affected by culture, for example, Herne). 50% of the German cities have a hemeroby value between 4.4 (Q_1_, examples: Wiesbaden, Detmold, Offenburg) and 5 (Q_3_, examples: Zwickau, Gütersloh, Kiel, Unna).

With respect to settlement density, degree of soil sealing and hemeroby, a dependence of the values on the size of the city (number of inhabitants) seems to be indicated. As expected, bigger cities have a higher proportion of soil sealing, have a stronger anthropogenic impact (hemeroby) and are more densely settled compared to cities with less than 100,000 inhabitants.

The statistical parameters of the indicators, calculated for all city districts (*n* = 144) of eight selected big cities, are represented in Table 3. The indicators for the accessibility and the provision of green space show a clearly differentiated range of values at the level of city districts. This shows that the city districts exhibit very different structural types (e.g., high-density centre versus low-density settlement with single-family homes or city districts with a high proportion of grassland). The mean degree of soil sealing amounts to 53.3% and does not fundamentally deviate from the values at the city level (cf. Table 2, mean degree of soil sealing for 187 German cities = 47.9%). The highest degree of soil sealing (max = 87.6%) is found in Munich-Westend (Schwanthalerhöhe). At the level of city districts, the average settlement density amounts to 245 inhabitants per ha and is, of course, higher than at the city level (cf. Table 2, mean settlement density of 187 German cities = 148.6 inh./ha). The mean degree of hemeroby points to a strong effect of culture or a strong anthropogenic impact (mean = 5.2). Upon a closer look at the individual city districts, it is noticeable that the city district Wiesbaden-Rambach is least affected by culture (min: i6 = 2.9). Frankfurt-Westend corresponds to the maximum value of 6.9. Moreover, Frankfurt-Westend has the highest density and overall the lowest supply or accessibility of green space (i1–i3).

### 3.2. Results of the Combined Indicator Application at City Level

**Figure 3:**
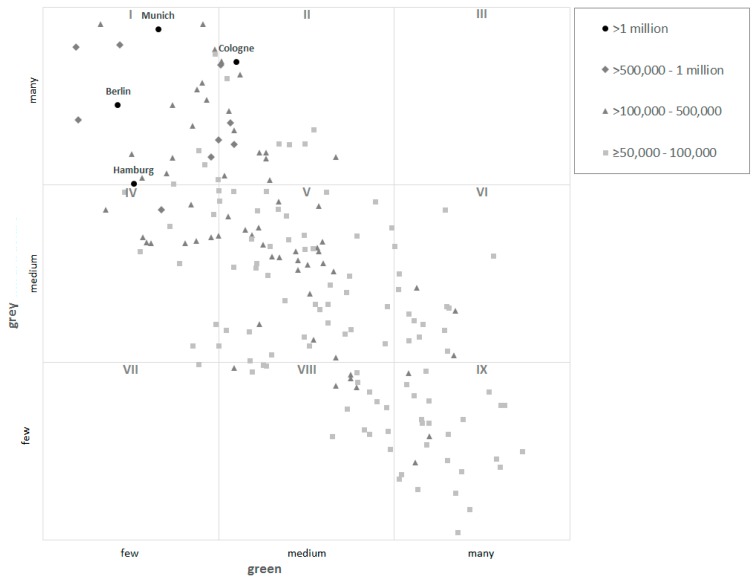
Distribution of the total number of points of 187 German cities >50,000 inhabitants, divided into classes of inhabitants, assessed with the combined indicator.

When the classification was carried out, it revealed that except for Cologne all cities in Germany with at least one million inhabitants exhibit much grey and little green characteristics (Figure 3). In big cities with many inhabitants, the grey characteristics also often predominate. Cities in North Rhine-Westphalia form an exception in this respect with a balanced level. The larger towns are represented in all occupied cells. However, they often exhibit a dominance of green characteristics. The class IX (little grey, much green) is almost exclusively occupied by larger towns (e.g., Passau, Bergisch Gladbach).

It is striking that hardly any cities appear in the cells III (much grey, much green) as well as VII (little grey, little green) (Figure 3). In the measurement approach used, indicator values are assessed based on ranks. The ranks are based on a comparison of indicator values with the values for other cities. It turns out that cities that exhibit a high indicator value for soil sealing, settlement density, hemeroby in the nationwide comparison do not simultaneously possess a high accessibility of green space relevant for recreation and provision with corresponding green spaces. The cities of Troisdorf in North-Rhine Westphalia (cell V) and Neubrandenburg in Mecklenburg-Vorpommern (cell VI) lie near the border to cell III. Both cities are characterised by a comparably high population density and soil sealing in predominantly inhabited settlement areas as well as larger forest, shrub and grassland areas close to the settlements. The cell VII is only occupied by the city of Unna in North Rhine-Westphalia. This is a larger town that exhibits a high share of farmlands and little green space relevant for recreation.

**Figure 4:**
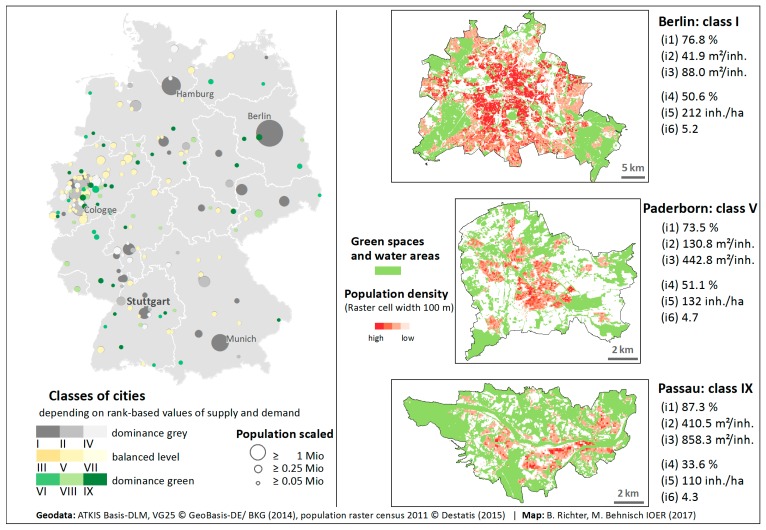
Cities categorised via ‘green/grey’ dominance and 3 examples of the cell types: I ‘many grey, few green’, V ‘medium grey, medium green’, IX ‘few grey, many green’.

A categorisation of the cities at the federal level, underpinned by three examples of typical representatives of types I (Berlin), V (Paderborn) and IX (Passau), illustrates the results in maps (Figure 4). It can be seen very clearly that cities with a high number of inhabitants and smaller compact cities are usually dominated by grey infrastructure and a strong human impact on urban nature. The city examples provide an insight, both visually and via city-wide parameters, into the situation of city types with the most representatives (60% of the 187 cities belong to the fields I, V and IX). The opposing trends of ‘grey” and ‘green’ characterisation with increasing or decreasing city size and number of inhabitants can be understood with a view to the listed parameters, population density and green space relevant for recreational, of the 3 representatives shown (Figure 4).

### 3.3. Results of the Combined Indicator Application at City-District Level

**Figure 5:**
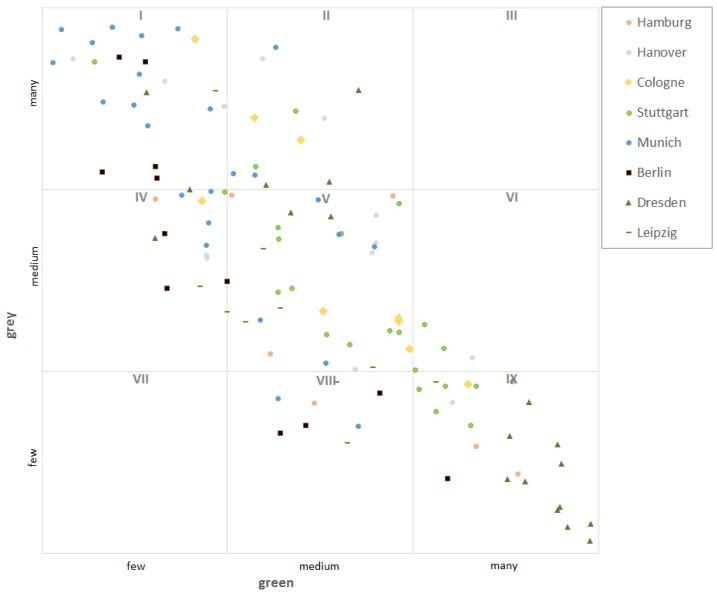
Distribution of the total number of points of 144 city districts assessed with the combined indicator.

While the eight big cities studied are characterised by ‘dominance of grey characteristics’ (Section 3.2), the distribution of the points for the 144 city districts assessed with the combined indicator reveals some differentiations (Figure 5). The city of Munich stands out with many city districts in cell I and no one in cell IX. As expected for the metropolis Berlin, its city centre is in cell I, while city districts in the periphery are in the cells III, VIII and IX. Hamburg and Cologne show a similar pattern. These cities of more than a million inhabitants have very complex qualities at the level of city districts. Stuttgart exhibits a particularly differentiated pattern, as city districts can be found in cells I, V, and IX. Many districts of Dresden are characterized as ‘green’ (cell IX).

### 3.4. Comparison of Results at City and City-District Levels within the Example of Stuttgart

**Figure 6:**
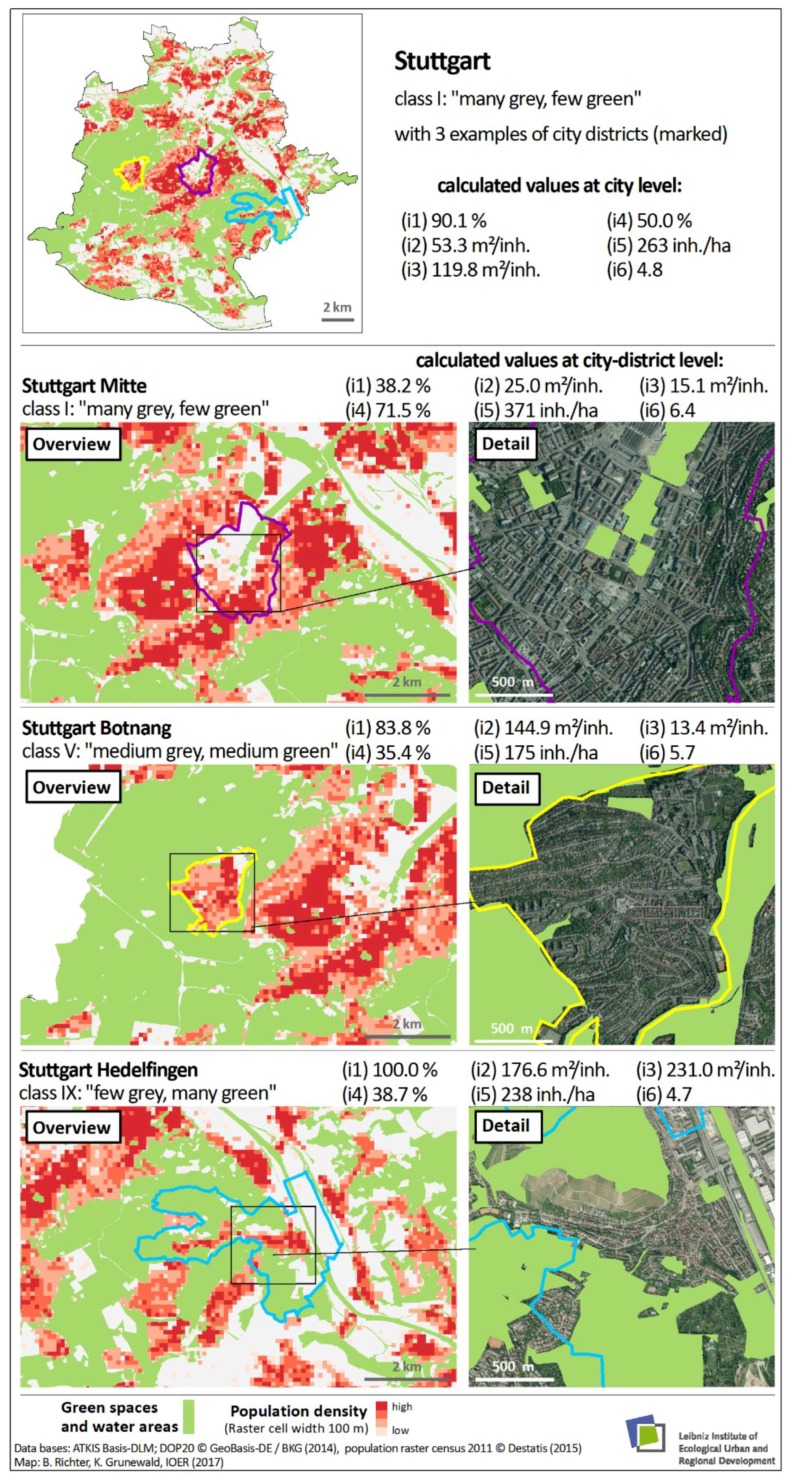
Overview of Stuttgart with 3 examples of city districts in the cells: I ‘many grey, few green”, V ‘medium grey, medium green”, IX ‘few grey, many green”.

Figure 6 shows the unequal distribution of urban green space within the example of the city of Stuttgart. The overview map at the top (entire city level) demonstrates that certain areas (dark red colour) are undersupplied with public urban green spaces (high population density and soil sealing); the other parts are ‘green’. This unequal distribution of urban green space within urban areas was found for a high number of major German cities (see also [60]).

For example, Botnang, a district of the city of Stuttgart, exhibits a high accessibility of green space (i1) and at the same time a very low provision with public green (i3). The degree of soil sealing (i4) there is comparable to that of the rather rural district of Hedelfingen, while the green space per inhabitant within the unit of investigation (i3) is comparable to that of Stuttgart Mitte. This shows that the city district of Botnang is a loose settlement with low density that is almost completely characterised by inhabited settlement areas and thus tends to exhibit a low need for public green spaces. This is also apparent from the classification result ‘balanced level’ (class V).

## 4. Discussion

A ‘green’ urban development figures prominently on the political agenda in Germany and other countries [13,38,39]. In this context, empirical data and action goals for ‘Green in the City’ based on indicators represent a basis for the pursuit of a more sustainable urban development, as green infrastructure has been stressed as an important factor for health and constitutes much of the quality of life in cities [15,24,26,48].

### 4.1. Specific Needs for Data and Indicators

Planners and policy-makers need objective and comparable measures and indicators reflecting urban green space provision across multiple communities. Such indicators should be evidence-based, unanimously defined, and universally applicable across various populations and environmental conditions. Green space characteristics refer to type, size, and quality of green spaces and the use functions that they allow [48]. Usually, the indicators are made operational in a GIS-based working procedure and take different dimensions into account.

Qualitative recommendations must complement quantitative benchmarks for urban green. Both are scale-dependent. Therefore, it has to be specified whether they concern an entire city (i.e., the entire urban green system within the administrative borders), one or more quarters of a city (city-district level) or an individual green space (site level). At each level, quantitative and qualitative aspects have to be taken into consideration [41,44].

Often, (unspecified) green space proportion indicators such as i2 and i3 measure the share of vegetated area in comparison to the entire city (e.g., [61]). If there are extended rural areas or larger forests within a reference area, the indicator value can be misleading, as the remote forests and fields give the appearance of a large extent of green space in an actually densely built-up city. Furthermore, indicator values depend sensitively on what kind of green space (only public green or private as well, only recreationally relevant or also agricultural land and wastelands) has been included in the calculation. The latter applies also to the ‘Green space per capita’ indicators. These provide a better insight into the possible demand for green spaces by the inhabitants [41]. The World Health Organisation (WHO) demands at least 9 m^2^/inh. green space in general and designates 50 m^2^/inh. as preferable [42].

### 4.2. Scores for the Presence of Green/Gray Characteristics

The six individual indicators, as well as the combined indicator used in this study, are rather simple, robust and reproducible measures. At a national level, they provide a comparison between the cities. Most of them are monitored via the IOER Monitor of Settlement and Open Space Development (www.ioer-monitor.de/home/?L=1), a permanent scientific service of the Leibniz Institute of Ecological Urban and Regional Development.

The indicator *(i1) ‘accessibility of urban green space’* shows that in 2015 nationwide 79.2% of the inhabitants of the cities studied were able to reach at least green spaces (>1 ha) at a linear distance of no more than 300 m (≈500 m walking distance) and larger green spaces (>10 ha) at a maximum linear distance of 700 m (≈1000 m walking distance). The result is easy to interpret, since the closer the degree of provision comes to 100%, the higher the welfare effect for the dwellers [62]. This target value is easier to justify, to compare and to communicate than the green-space area per inhabitant. The number of inhabitants is more useful as a reference quantity for examining the accessibility of green spaces than the municipal area, since a concentration of population has a stronger influence on the indicator, which therefore exhibits a closer relationship to the people looking for rest and recreation.

The distances and green space categories for (i1) were stipulated/chosen by the authors and planners in the framework of a research project [50,56]. In principle, they are in line with the recommendations for primary and secondary indicators of green space provision given by the WHO 48]. However, it only makes sense to compare numbers if they have been calculated with similar methodology and comparable databases. For instance, the study in Flanders uses 400 m/800 m linear distances [63], while in the UK, urban dwellers should have access to 2 ha of green space within a 300 m straight-line distance from the place of residence [64].

Wüstemann et al. [60] used the European Urban Atlas and not ATKIS Basis-DLM as a data base for assessing German cities. They estimated that 93% of the German households have access to green spaces within 500 m and 74.1% within 300 m linear distance from the location. The green-space provision for major German cities was calculated to be 8.1 m^2^ per capita (median). Kabisch et al. [47] show that the share of the population in European cities living within 500 m linear distance from green and forest areas with a minimum size of 2 ha ranges from 11% to 98%.

Green space accessibility considers not only the available green space but also how to get there. It can be assessed using linear distance or actual path length [48,59]. These indicators are more reliable regarding city boundary effects, as green space at a larger distance is not considered regardless of whether it belongs to a municipal area or not. Therefore, the indicator will not be affected by incorporation of villages and other types of changes in the city’s area [41].

Adding three more indicators (i4, i5, i6) complements the provision of green space with proxies for the grey characteristics and human impact on urban nature (hemeroby). An approximately linear relationship between cities or city districts characterised as ‘green’ and such characterised as ‘grey’ can be discerned (Figure 3 and Figure 5). This approach has several limitations, because the sub-indicators are not independent among themselves and the assignability, for instance of hemeroby (i6) to ‘grey’ needs further investigations.

### 4.3. Results are Useful for Communication

The interpretation of the results is supported by deriving a ranking combined with statistical quantities. In this way, city types can be identified by analysing the indicators of ‘green’ (accessibility and provision of green space and the potential demand situation). With respect to the indicators, all cities exhibit a certain profile of green-space provision and need situation, i.e., they can be assigned to a certain type of provision of green space. With the help of the indicators used, structural aspects and connections between provision of green space and ‘grey’ aspects (soil sealing, settlement density, hemeroby) can be differentiated further. This reflects the spatial heterogeneity and properties of urban ecosystem service supply and demand [15,42,44,47].

The approach used for categorising cities is a practical approach for a combined evaluation of several parameters and helps when discussing the calculated indicators. Thus, big cities sometimes exhibit high values for the accessibility of green space, whereas mostly cities with fewer inhabitants and a large area attain high indicator values for the provision of green space. This shows that the results for green-space provision (particularly for i3) are strongly influenced by the area size and implemented reforms of administrative regions. Cities which have incorporated surrounding municipalities are thus characterised by more green space per inhabitant, since newly added areas mostly exhibit lower population densities and large proportions of free space. By contrast, municipalities that mainly consist of a core city have a lower provision of green space. These problems do not occur with approaches for the accessibility of green space, since areas with high population density and green spaces in the surroundings influence the indicator value more strongly than e.g., areas outside the core city with a lower number of inhabitants.

A representative example (Stuttgart) was used to compare how the different indicator values turn out at the levels of city and city districts (Section 3.4, Figure 6). Overall, Stuttgart represents a municipality with relatively high accessibility of green space and medium provision of green space per inhabitant. At the level of city districts, the indicator can describe the provision with public green space per inhabitant on a smaller scale and can better indicate possible deficits, which provides important cues for the concrete planning of re-densification or green-space protection. But the data used in our study are in most cases not suitable for the neighbourhood/land owner scale.

### 4.4. Reflexion of the Methodology

In the context of the selected methodology, the question of further opportunities for study arises with a view to, on the one hand, inspecting indicators of urban green provision in more depth with respect to their spatial, temporal, and statistical characteristics and on the other hand, conducting a multi-dimensional analysis and an assessment with the aim of gaining new and useful insights about the indicators and their relationship structures.

The term ‘Urban Data Mining’ characterises a methodology developed for the urban context which serves to discover logical or mathematical and sometimes complex descriptions of patterns and regularities in data sets and to use them to derive and assess findings [65,66]. Classification procedures for an in-depth analysis are suitable in the context of assessing the provision of green space in cities. Inductive generalisations about the objects of study are made by finding a common term for designating the grouped phenomena which are similar in certain aspects or only differ insignificantly. In the present approach, this has been implemented using the 9-cell matrix (portfolio analysis) for categorising the objects of study according to the aggregated criteria for characterising urban green space provision.

In addition to the applied rank-based classification approach it is possible to use extreme value standardization for each indicator [67]. Regarding the normalisation of extreme values, the individual indicators can be transformed into values between zero (unfavourable value) and one (favourable value). The total index for the city is then the sum of all inflowing indices, which is subsequently normalised. The higher the total index for a spatial unit, the higher the quality of the interdependency.

In future, depending on the availability of further data, the set of variables used can be supplemented, the classification methodology can be compared (e.g., cluster analysis versus ranking procedure), and changes in land use can be characterised in a multi-dimensional way (dynamic perspective). Moreover, a distinction is to be made between descriptive analyses that look for groups (cluster) and predictive analyses that are more interested in patterns of connections (link), temporal patterns (sequence), rules and dependencies as well as formulas and regularities.

The Modifiable Areal Unit Problem (MAUP) needs to be considered when investigating the green space provision of cities and interpreting the results. In correspondence to any spatial analysis, we suggest computing the multi-indicator at different spatial scales. For example, in-depth studies can take place at much finer spatial scales in order to better understand the characteristics for cities of approximately the same sizes [44]. We see future applications both at the administrative level and the grid level.

## 5. Conclusions

Indicator-based assessments of the green spaces are necessary for supporting political decision-makers, planners and practitioners for ecologically sustainable urban development (e.g., [13,45,48,68,69]). Individual measures represent individual aspects with concrete spatial and temporal reference. A ranking of cities, for example for the ‘Greenest City of Germany’ (‘Grünste Stadt Deutschlands’, [61]), or a comparison of the green space performance of cities (e.g., between Chinese and German cities, [15]), is often problematic, since calculations can be carried out in very different ways. But it fosters the debate on ‘Green Urban Development’.

For any indicator, the result depends on how the individual parameters are set. During the development of the indicators, this includes e.g., statistical bases, area sizes, land use types, distances and reference areas. The measurement approach presented here allows a comparative characterisation of the provision of inhabitants with green space in the areas studied, since the indicator values and the classification result are based on a uniform methodology.

The tested approach, which is based on a combination of indicators for describing grey and green characteristics, allows a more aggregated characterisation of the units of analysis with respect to the provision of inhabitants with public green space. The classification was carried out using a rank-based point scheme. In principle, this approach could be applied in cities all over the world. In future, points could be awarded e.g., based on threshold values (e.g., minimum green space size), or further classification methods could be tested (e.g., cluster analysis).

However, it is rarely possible to represent a topic completely using one indicator or even an index, as a compromise needs to be struck between the complexity of the representation of the object of study and the comprehensibility and communicability of the results [70]. The generalisation in applying models is typical and needs to be taken into account in interpreting the results.

It should be critically noted that the 3 selected indicators for green spaces essentially only characterise the provision of the population with public green spaces (e.g., accessibility and provision of green spaces), but not the actual characteristics of all green spaces in the settlement area. It is obvious that small pocket gardens are also important for the connectivity of green spaces and the biodiversity. In case of better data for the entire of Germany it might be very interesting to take into account all green spaces and to judge about the size, characteristic and quality of public and non-public green spaces. It might be also interesting to analyse co-localisation patterns of green spaces that link the coexistence of a number of non-spatial features in a spatial neighbourhood. Such additional information is valuable both for the practitioner and the data analyst and forms the basis for broader indicator concepts in the future.

The measurement approach presented allows a classification, in this study shown for the biggest German cities, according to their provision of green and grey characteristics (dominance green or grey or balanced level, cf. Figure 3 and Figure 4). For a detailed analysis and interpretation of the supply situation in individual municipalities, it turns out to be useful to look at individual indicator values and to carry out an analysis at the level of city districts or the grid level.

Spatial and urban planning should secure areas for both green and grey infrastructure to a balanced extent and define them in terms of planning. The individual measures of discussed green and grey characteristics can contribute to providing the municipal decision-makers and planners with a basis for argumentation in order to give weight to the goals of a need-oriented, interconnected provision of green space, relative to other goals of urban development [13,44,54]. The applicability or usefulness of the ‘combined indicator’ for city monitoring cannot yet be conclusively assessed. The questions that need to be answered for this purpose include whether the multi-indicator yields stable results when several points in time are analysed, how changes are recognised, and how the movement of the objects in the matrix can be observed/compared.

## Figures and Tables

**Table 1:** Overview of implemented measures.

Indicator Name (Short Name)	Formula	Linear Distance (Approximate Path Distance)	Included Object Types (ATKIS Basis-DLM)	Reference Value
accessibility of urban green space (i1)	$\left(i1\right)=\frac{{\mathrm{NOI}\;}_{300\;m|700\;m\;\mathrm{dist}.\;\mathrm{green}\;\mathrm{areas}\;(\geq 1\;\mathrm{ha}|\geq 10\;\mathrm{ha})}}{\mathrm{population}}\times 100$	≤300 m (500 m) and ≤700 m (1000 m)	recreational land use types: green areas, parks, cemeteries, grassland, orchard meadows, forests, woods, surface waters	population (population grid)
	*NOI* number of inhabitants			
green-space provision—settlement (i2)	$\left(i2\right)=\frac{{\mathrm{green}\;\mathrm{area}\;}_{300\;m\;\mathrm{dist}.\;\mathrm{to}\;\mathrm{ISA}}}{\mathrm{population}}\times 100$	≤300 m (500 m)		
	*ISA* inhabited settlement areas			
green-space provision—total (i3)	$\left(i3\right)=\frac{{\mathrm{green}\;\mathrm{area}}_{\mathrm{total}}}{\mathrm{population}}\times 100$	-		population (municipal directory information system)
soil sealing—settlement(i4)	$\left(i4\right)={\mathrm{Imperviousness}\;}_{\mathrm{ISA}}$ ^1^	-	residential and mixed use areas	inhabited settlement area
	*ISA* inhabited settlement areas			
settlement density (i5)	$\left(i5\right)=\frac{\mathrm{NOI}}{\mathrm{ISA}\times 10000^{-1}}$	-	residential and mixed use areas	
	*ISA* inhabited settlement areas			
	*NOI* number of inhabitants			
hemeroby (i6) is based on Walz & Stein (2014)	$\left(i6\right)=\overset{n}{\underset{h=1}{\sum }}f_{n}\times h$	-	all land use types, grouped by degree of hemeroby	reference area
	*N* number of degrees of hemeroby (*n* = 7)			
	$f_{n}$ proportion of hemeroby *n*			
	*H* degree of hemeroby			

^1^ Imperviousness Layer 2012 of European Environment Agency (EEA) in 100 m grid based on Copernicus Land Monitoring Services: http://land.copernicus.eu/pan-european/high-resolution-layers/imperviousness. ATKIS: Authoritative Topographic-Cartographic Information System; Basis-DLM: Digital Basic Landscape Model.

**Table 2:** Overview of statistical parameters for the calculated measures in German cities (≥50,000 inhabitants, *n* = 187, classified by number of inhabitants).

**Parameter Fruppiert Nach Stadtgrößen**	**Min**	**Q_1_**	**Median**	**Mean**	**Q_3_**	**Max**	**StDev.**
i1 accessibility of urban green space (%)	49.5	71.1	80.4	79.2	87.5	99.1	10.9
i2 green space provision—settlement (m^2^/inh.)	36.1	89.6	128.7	148.3	181.7	902.4	95.5
i3 green space provision—total (m^2^/inh.)	46.1	206.8	346.8	453.9	589.0	1942.2	365.6
i4 soil sealing—settlement (%)	23.4	43.6	48.4	47.9	51.9	66.9	7.1
i5 settlement density (total) (inh./ha)	73.0	114.5	138.0	148.6	172.5	317.0	47.9
i6 hemeroby (class)	3.5	4.4	4.7	4.7	5.0	5.5	0.4
**Cities ≥ 500,000 inhabitants (*n* = 13)**	**Min**	**Q_1_**	**Median**	**Mean**	**Q_3_**	**Max**	**StDev.**
i1 accessibility of urban green space (%)	59.9	72.1	76.8	78.3	88.7	94.6	11.1
i2 green space provision—settlement (m^2^/inh.)	36.1	53.3	62.4	67.4	89.9	108.9	23.8
i3 green space provision—total (m^2^/inh.)	46.1	98.8	119.8	130.7	127.5	251.1	55.9
i4 soil sealing—settlement (%)	47.2	50.2	53.9	54.4	58.2	152.0	5.1
i5 settlement density (total) (inh./ha)	140.0	178.0	211.0	209.8	242.0	317.0	51.7
i6 hemeroby (class)	4.8	5.1	5.1	5.1	5.2	5.5	0.2
**Cities ≥ 100,000 inhabitants (*n* = 64)**	**Min**	**Q_1_**	**Median**	**Mean**	**Q_3_**	**Max**	**StDev.**
i1 accessibility of urban green space (%)	52.9	72.6	81.2	79.7	87.4	98.9	10.7
i2 green space provision—settlement (m^2^/inh.)	41.3	79.9	94.3	116.0	151.4	271.3	54.1
i3 green space provision—total (m^2^/inh.)	74.0	164.5	252.4	289.6	363.9	676.9	159.2
i4 soil sealing—settlement (%)	34.2	46.9	49.6	49.7	52.9	66.6	6.1
i5 settlement density (total) (inh./ha)	87.0	134.8	157.5	166.0	184.8	291.0	47.0
i6 hemeroby (class)	3.9	4.6	4.9	4.8	5.1	5.5	0.4
**Cities ≥ 50,000 inhabitants (*n* = 110)**	**Min**	**Q_1_**	**Median**	**Mean**	**Q_3_**	**Max**	**StDev.**
i1 accessibility of urban green space (%)	49.5	70.6	80.4	79.0	87.3	99.1	11.2
i2 green space provision—settlement (m^2^/inh.)	45.0	113.6	145.7	176.2	207.1	902.4	107.8
i3 green space provision—total (m^2^/inh.)	83.9	297.0	440.3	587.7	771.4	1942.0	408.3
i4 soil sealing—settlement (%)	23.4	41.5	46.1	46.0	50.5	66.9	7.1
i5 settlement density (total) (inh./ha)	73.0	103.5	127.0	131.2	149.2	254.0	37.7
i6 hemeroby (class)	3.5	4.3	4.7	4.6	4.8	5.3	0.4

Q_1_: first quartile; Q_3_: third quartile; StDev.: standard deviation; inh.: inhabitants.

**Table 3:** Overview of statistical parameters for the calculated measures at the level of city districts (studied cities: Berlin, Cologne, Dresden, Hamburg, Hanover, Leipzig, Munich, Stuttgart).

Parameters	Min	Q_1_	Median	Mean	Q_3_	Max	StDev.
i1 accessibility of urban green space (%)	0.6	59.5	73.5	70.6	88.0	100.0	23.1
i2 green space provision—settlement(m^2^/inh.)	0.4	42.7	77.3	168.3	116.5	3231.5	347.4
i3 green space provision—total (m^2^/inh.)	0.1	41.3	94.1	297.7	248.5	3585.7	594.0
i4 soil sealing—settlement (%)	20.6	42.9	53.4	53.0	60.4	87.6	13.7
i5 settlement density (total) (inh./ha)	28	163.8	213.5	244.8	297.5	972.0	132.9
i6 hemeroby (class)	2.9	4.8	5.3	5.2	5.7	6.9	0.7
